# Hydrogels for Bone Regeneration: Properties, Additives, Preclinical and Clinical Applications

**DOI:** 10.3390/gels11110914

**Published:** 2025-11-16

**Authors:** Nesya Graupe, Saliha Ahmad, Ahmad Zia, Michael Hadjiargyrou, Azhar Ilyas

**Affiliations:** 1 Bio-Nanotechnology and Biomaterials (BNB) Lab, New York Institute of Technology, Old Westbury, NY 11568, USA; ngraupe@nyit.edu (N.G.); sahmad27@nyit.edu (S.A.); 2 College of Osteopathic Medicine, New York Institute of Technology, Old Westbury, NY 11568, USA; 3 Department of Bioengineering, New York Institute of Technology, Old Westbury, NY 11568, USA; 4 Virginia Cancer Specialist, Fairfax, VA 22031, USA; ahmad.zia@usoncology.com; 5 Department of Biological and Chemical Sciences, New York Institute of Technology, Old Westbury, NY 11568, USA; mhadji@nyit.edu; 6 Department of Electrical and Computer Engineering, New York Institute of Technology, Old Westbury, NY 11568, USA

**Keywords:** hydrogels, bone regeneration, fracture repair, tissue engineering, biocompatibility

## Abstract

Severe bone loss from trauma, fractures, tumor resections, and disease are devastating injuries that do not heal completely without external, and most of the time surgical, interventions. Although surgical interventions such as bone grafts and metal prostheses are commonly employed, these conventional approaches present several limitations, including limited donors, risks of immune rejection and postoperative inflammation, and significant pain experienced by both donors and recipients. Hydrogels offer a promising alternative because of their controllable mechanical properties, biocompatibility, and structural resemblance to the extracellular matrix. In addition, hydrogels can be modified with substances such as growth factors, hormones, and drugs to facilitate accelerated bone repair. This review summarizes the recent advances in hydrogel development for bone repair, their structural design, biological functionality, and preclinical and clinical applications.

## 1. Introduction

Fractures constitute a break or crack in the bone that occurs because of trauma, illnesses, aging, or congenital defects. Approximately 178 million new bone fractures were recorded in 2019, which represents a 34% increase in injury prevalence since 1990 [1]. The conventional treatments for these injuries are autografts, allografts, and xenografts, but are associated with several shortcomings, including disease transmission risks, a lack of donors, infection, and host rejection [2]. Tissue engineering offers an alternative to these highly invasive bone injury treatments. Specifically for bone, the major goal is to create either natural or synthetic scaffolds that can provide structural support and allow for cell adhesion, migration, proliferation, and differentiation to restore the injured tissue and, more importantly, provide functionality. Ultimately, the newly generated tissue should match the biomechanical properties of intact bone to withstand the demands of loading forces that may arise from daily use [3].

Hydrogels are three-dimensional (3D) hydrophilic polymer networks that can retain large amounts of water while keeping their structural integrity. The first hydrogels for biological use were developed in 1960 by Wichterle and Lim, who demonstrated that it was possible to develop a stable, biocompatible synthetic hydrogel for experimental use [4]. Hydrogels have an excellent biocompatibility, an ability to have a slow and controlled release of loaded biomolecules and drugs, and a porous structure that promotes gas exchange and nutrient/waste transfer between their microenvironments [5]. Hydrogels can be functionalized with molecules such as genes, proteins, drugs, and cells to accelerate their applicability and efficacy [6]. Hydrogels have a network structure that can promote the proliferation and adhesion of osteoblasts [7]. In addition, they have a similar structure to the extracellular matrix (ECM) of bone and cartilage, which creates a favorable microenvironment for cells [8].

**Figure 1:**
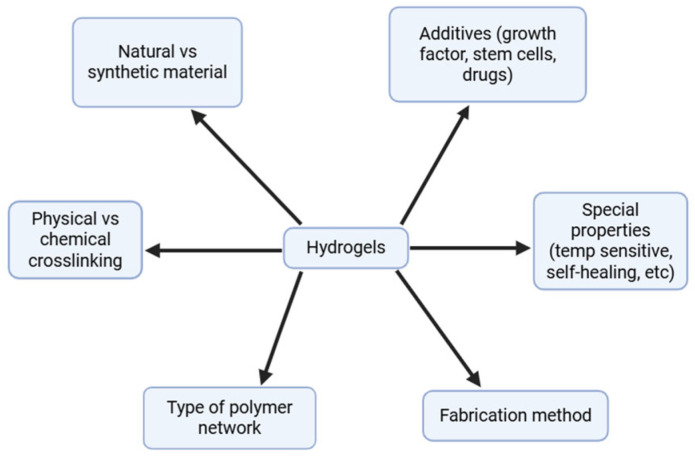
Hydrogel properties that can be fine-tuned based on their application. Created in BioRender (https://BioRender.com/).

Hydrogels offer a promising treatment for bone defects because they can fit any defect shape and size and are biodegradable and integrate well with the surrounding tissue, thus avoiding the need for surgical removal and reducing the inflammatory response [9]. Hydrogels have a high swelling ratio which allows them to absorb wound exudate and reduce tissue infection. With all their advantages (Figure 1), hydrogels have the potential to become a therapeutic reality for the treatment of large bone defects due to their use as scaffold materials for drug and growth factor delivery and a broad range of other biomedical applications [10].

This manuscript explores the use of hydrogels in bone repair, new developments, their limitations, and their future applications. The literature search for this review utilized the public databases PubMed and Google Scholar, using the following terms: “*hydrogels for bone repair*”, “*hydrogel clinical applications bone repair*”, and “*hydrogels bone regeneration growth factors*”. Ten to fifteen recent and foundational representative manuscripts were then selected to be included for each of these topics, growth factors in hydrogels, other hydrogel additives, special hydrogel properties, preclinical applications, and clinical applications, as outlined in this review.

## 2. Properties of Different Types of Hydrogels

### 2.1. Hydrogel Fundamentals

Hydrogels are constructed from natural biomaterials or synthetic materials that have components with hydrophilic surfaces that allow cells to adhere, migrate, proliferate, and differentiate, but a have poor stability [11]. Natural biomaterials include gelatin, hyaluronic acid, alginate, chitosan, and dextran. Gelatin scaffolds are made from denatured collagen and hyaluronic acid, molecules found in human connective tissue. Chitosan, like hyaluronic acid, is found in the external connective tissue of crustaceans. Alginate is a thick, stable polymer found in brown seaweed. Dextran is complex sugar derived by bacteria that use sucrose as a source. While these materials are derived from living organisms and can mimic the matrix (ECM), due to their variability, poor mechanical quality, and difficulty in sterilization, they are challenging to use.

On the other hand, synthetic biomaterials are controllable and reproducible [12], but they have a lower biological activity [13] and poor safety and biocompatibility. Some common synthetic biomaterials include PEG (polyethylene glycol), pol(N-isopropylacrylamide), polycaprolactone, PGA (polyglycolic acid), PLLA (polylactic acid), PLGA (poly (lactic-co-glycolic acid)), PVA (Polyvinyl alcohol), and polypropylene fiber. To increase biocompatibility and reduce the immune response, a hybrid combination between both natural and synthetic materials can leverage the benefits of each, providing a composite scaffold for bone fracture repair [14,15].

**Figure 2:**
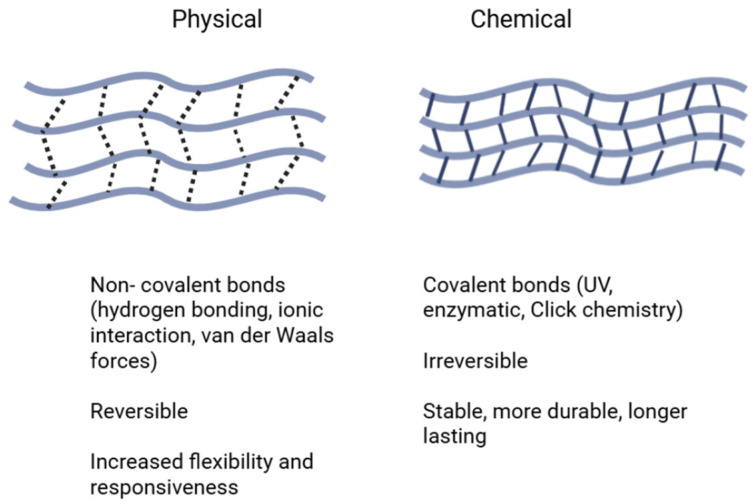
Comparison between physical and chemical crosslinking of polymer chains in hydrogels. Created in BioRender (https://BioRender.com/).

Hydrogels have specific mechanical properties dependent on their crosslinking type, which strengthens the hydrogel network and allows them to retain their elasticity and shape. When implanted in vivo, these scaffolds need to retain their structure while they degrade over time. Mechanical strength is dependent on the density and crosslinking of the polymers [16], and both natural and synthetic hydrogels are usually crosslinked physically or chemically to produce strong tissue engineering scaffolds that are stable 3D networks (Figure 2). The crosslinking degree of the hydrogel directly affects its density [17]; an increased crosslinking density creates a tighter polymer network structure. Physical crosslinking relies on weak intermolecular forces and uses crystallization techniques and ionic, electrostatic, or hydrophobic interactions [18]. In contrast, chemical crosslinking involves creating permanent covalent bonds, leading to a greater network stability. Hydrogels can be crosslinked chemically by free radical polymerization, click chemistry, enzymatic crosslinking, photo-crosslinking, or with the use of epoxy or aldehyde. Chemical crosslinking also has greater control over flexibility and spatiotemporal accuracy [19]. Furthermore, hydrogels can also be complexed with ceramics such as calcium phosphate, fumed silica nanoparticles, bioactive glass nanoparticles, and nano-hydroxyapatite, offering different biomechanical properties for these composite materials [20].

Four different types of polymer networks are used in hydrogels: homopolymeric, copolymeric, semi-interpenetrating network (semi-IPN), and interpenetrating polymeric (IPN). Homopolymer hydrogels are polymerized by a single hydrophilic monomer [21], while copolymeric hydrogels have two or more different monomers with at least one hydrophilic component to prevent swelling [22]. Semi-IPN hydrogels have one linear polymer that penetrates another crosslinked network with no other chemical bond between the two [23], while IPNs are formed by two or more polymer networks that are fully interwoven but not covalently bound to each other [23]. Semi-IPN hydrogels have a greater flexibility and adaptability and are more suitable for controlled swelling, while IPN hydrogels have a higher mechanical strength, durability, and are used frequently for tissue engineering and drug delivery applications [24].

Hydrogel scaffolds can be designed and custom-manufactured into specific shapes and sizes based on patient-specific needs. This is performed using methods such as microfluidics, emulsion, and nano-molding techniques by controlling the gelation conditions or processing parameters [25]. In addition, there are different 3D printing techniques that can be used to create hydrogel-based scaffolds, namely extrusion-based, inkjet-based, and laser-assisted bioprinting [26]. Extrusion printing uses a computer-controlled layer through the layer deposition of molten/semi molten polymers, pastes, dispersions, or polymer solutions with a movable nozzle, allowing for the controlled deposition of bioink materials on a stationary surface [6]. Inkjet printing uses digital data from the computer and reproduces it on a substrate through ink droplets and is suitable for complex scaffolds. Laser printing consists of stereolithography and two-photon polymerization and works through the sequential deposition of light energy in predefined patterns. Using these methods, hydrogels can be easily fabricated into 3D scaffolds at a low cost, with the ability to spatially pattern cells and biomolecules, as well as incorporate bioceramics for accelerated fracture repair [6,20,27]. And, lastly, controlling the generation of porous hydrogels designed for cell infiltration and migration is crucial for tissue regeneration.

### 2.2. Hydrogel Scaffold Considerations for Bone Repair

Osteoconduction is the ability to form new bone on the surface of biomaterials, and it supports the migration, proliferation, and differentiation of bone progenitor cells, angiogenesis, matrix deposition, and calcification [28]. Hydroxyapatite and tertiary calcium phosphate are two biomaterials that have shown good osteoconductivity because they are similar to the natural bone matrix [29]. Osteoconduction leads to osteogenesis via proliferation, growth, and the maturation of osteoprogenitor cells into osteoblasts, promoted by osteoinductive factors released by various surrounding cell types. Osteoinductive factors, nutrients, and oxygen are all necessary for osteogenesis as well, and can also lead to angiogenesis. The ideal bone scaffold should be biocompatible, non-toxic, and non-immunogenic, with pores that allow for the migration of endogenous cells, transport of nutrients, waste removal, and growth of blood vessels and nerves. Together, they will support the scaffold in the immunological environment surrounding the scaffold.

There are several different ways that hydrogels can be applied in vivo, including percutaneous injections, preformed scaffolds, photopolymerizable systems, and coatings on implants. In injectable hydrogels, liquid precursors are percutaneously injected into the defect and undergo gelation in situ, which enables them to fill irregular cavities with minimal surgery. Stadelmann et al. injected osteoporotic rats with a hydrogel containing HA2, HA2-ZOL, or NaCl control and showed that the treatment improved bone density and structure [30]. Preformed hydrogel scaffolds are usually fabricated ex vivo and then implanted during surgery [31]. Photopolymerizable scaffolds contain photoinitiations that activate in response to UV or visible light and undergo rapid crosslinking [32]. Lastly, orthopedic hardware can be modified with a thin hydrogel layer to serve as a reservoir for osteoinductive factors, drugs, or cells, or to discourage the formation of a biofilm to improve osseointegration and local bioactivity [33]. Zhang et al. created a nanozyme-reinforced photodynamic hydrogel to combat biofilm infection [34].

### 2.3. Biofunctionalization Strategies

**Figure 3:**
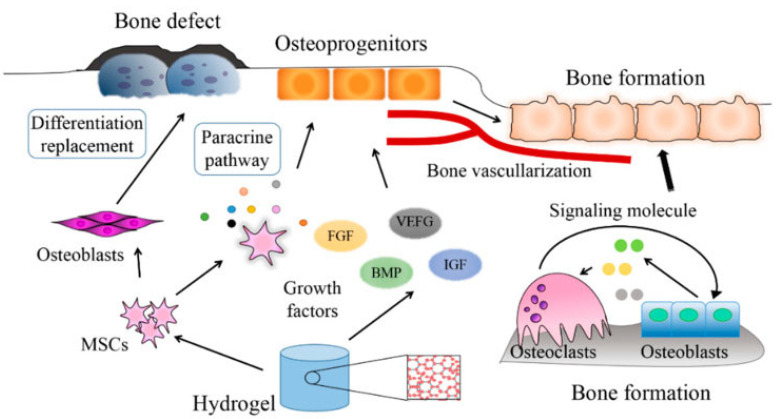
Mechanisms by which mesenchymal stem cells and growth factors are used in repair of damaged bone tissue [42].

The addition of growth factors in hydrogels represents one of the most applicable approaches. Growth factors are proteins that are essential in stimulating cell growth and promoting osteogenesis and bone repair. Growth factors can be incorporated into hydrogels to stimulate molecular bioactivity, since they absorb water, swell, and mimic the natural ECM [3]. Furthermore, hydrogels can be designed to release these bioactive factors in a controlled manner, allowing for the continuous formation of bone matrix and vascular networks [35]. Examples of such growth factors include Bone Morphogenetic Proteins (BMP-2, BMP4, BMP-7), Insulin-like Growth Factor (IGF), Fibroblast Growth Factor (FGF), Vascular Endothelial Growth Factor (VEGF), and Platelet-derived Growth Factor (PDGF) (Figure 3) [35,36].

BMPs are a class of cytokines that serve as signals for stem cells, signaling them to induce new bone formation. BMP-2, BMP-4, and BMP-7 are key in the differentiation of mesenchymal stem cells into osteoblasts [37]. IGF regulates cellular growth [38]. These factors communicate with various cell types to regulate bone activity, growth, and remodeling. FGFs represent a family of proteins that control the balance between bone formation, differentiation, and apoptosis [39]. VEGF is a potent factor that is responsible for angiogenesis, which is necessary for the delivery of the nutrients and oxygen necessary for successful bone development and repair [40]. Lastly, PDGF is a key protein that also aids in the wound healing process. It can inhibit bone formation as well as promote it [41], depending on the microenvironment. As such, integrating these growth factors into various hydrogels will promote the healing of fractured bones.

## 3. Results for Tested Hydrogels Loaded with Additives

Hydrogels can be loaded with a wide variety of additives, including stem cells, exosomes, and nanoparticles, and tested in various animal models or ex vivo. A variety of animals models are used to examine the scaffold’s ability to mimic the ECM and exert its effects on tissue repair.

### 3.1. Hydrogel Testing on Rat Models

Several studies have integrated additives, stem cells, and exosomes that are loaded into hydrogels, and their applicability is assessed in rat models. Lu et al. [43] loaded human urine-derived stem cell exosomes into gelatin methacrylate and hyaluronic methacrylate hydrogels and injected these into defects of the cranial bone in a rat model. They showed that hydrogel promoted the osteogenesis of bone marrow mesenchymal stem cells and angiogenesis of endothelial progenitor cells by comparing bone volume/tissue volume and trabecular thickness between the groups. Yang et al. [44] engineered exosomes that were enriched with BMP-2 and loaded them onto a gelatin methacryloyl (GelMA) hydrogel. Exosomes were slowly released, which resulted in a prolonged effect of BMP-2 endocytosed by the recipient cells, displaying a greater capacity for bone regeneration in a rat calvarial defect model.

**Figure 4:**
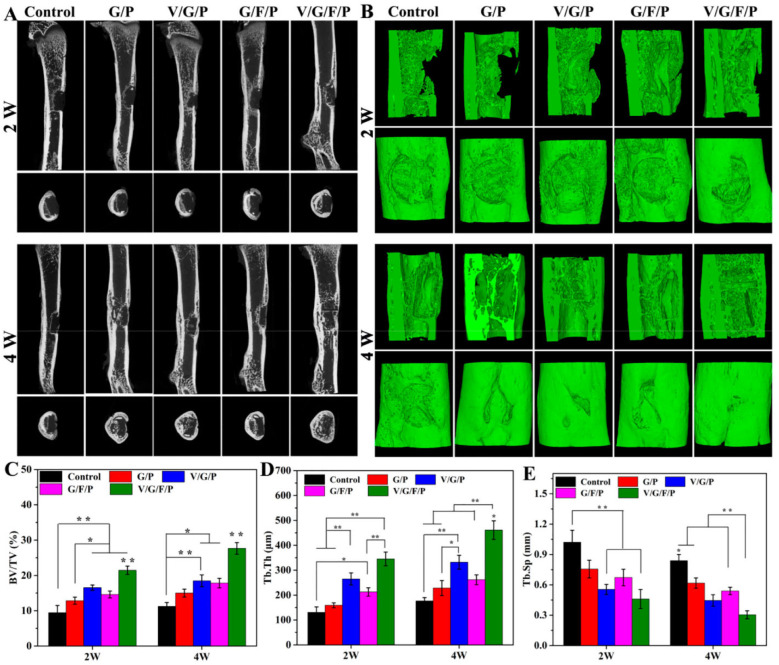
Micro-CT analysis of in vivo bone regeneration 2 weeks and 4 weeks after surgery. (**A**) Two-dimensional coronal and axial views of the femur defect area; (**B**) regenerated bone within defect; (**C**–**E**) show a quantitative analysis of bone formation. * *p* < 0.05, ** *p* < 0.01, compared to the other groups. Adopted from [47].

Nanoparticle integration into hydrogels is another approach using rat models. Huang et al. [45] developed a poly(L-glutamic acid)-g-Tyramine/VEGF/Strontium-doped Borosilicate nanoparticle that had the capacity of hydroxyapatite formation, mineralization, and gradual release of essential functional ions and VEGF. It was shown that it promoted the proliferation and osteogenic differentiation of rat bone marrow mesenchymal stem cells and improved the tube-forming capability of human umbilical vein endothelial cells. Following implantation into a rat calvarial defect, it promoted robust bone repair. Hu et al. [46] loaded super-paramagnetic iron oxide nanoparticles onto gelatin sponges, implanted the scaffold into the fresh incisor sockets of rats, and showed an enhanced bone regeneration and a greater formation of new bone without applying an external magnetic field. The microcomputed tomography (µ-CT) data showed that the SPIONs-GS group had more newly formed bone (64.44 ± 10.92 vs. 28.1 ± 4.49, *p* < 0.0001) and an alveolar ridge that was better preserved at 4 weeks (0.962 ± 0.01 vs. 0.92 ± 0.01, *p* < 0.0001) than the blank control group. Zhang et al. [47] created a dual-drug delivery scaffold system that consisted of a 3D-printed PCL scaffold containing mesoporous silica nanoparticles with the drug fingolimod (for structural support and to promote angiogenesis/osteogenesis) and a vancomycin-loaded hydrogel from aldehyde hyaluronic acid and carboxymethyl chitosan. The authors reported that the composite scaffold had concentration-dependent antimicrobial properties and promoted infection control and bone regeneration in a rat femoral drill hole model (Figure 4). Zhou et al. [48] developed a nanocomposite hydrogel powered by ultrasound to electrically accelerate irregular bone defect healing by crosslinking amine-modified piezoelectric nanoparticles and biopolymer hydrogel networks. They exhibited that, in response to ultrasound radiation, the hydrogel could generate a controllable electrical output of −41.16 to 61.82 mV, which enhanced the osteogenic effect and was validated in a rat calvarial critical-size defect model. Bhushan et al. [49] tested the biocompatibility of gelatin–chitosan hydrogels loaded with cerium oxide nanoparticles, a material that can scavenge free radicals. They found that the scaffolds are highly biocompatible, displaying an 89% porosity, a compressive modulus of 178.25 kPa, and a 1 calcium/phosphorous ratio of 1.83.

### 3.2. Hydrogel Testing on Other Animal Models

Hydrogels have also been tested on other animal models and offer different benefits. Bi et al. [50] created a chitosan and poly(vinyl) alcohol hydrogel that demonstrated a tensile strength of 0.24 MPa, elongation break at 286%, and compressive strength of 0.11 MPa at a strain of 60%. Surface mineralization by the introduction of hydroxyapatite gave the hydrogel the ability to induce rat bone marrow stem cell differentiation, an indication that it has the potential to be a bone repair material. Mussel-inspired hydrogels offer significant functional advantages for bone and cartilage regeneration, notably enhancing the efficacy of stem cells in these applications [51]. A mussel-inspired strategy was developed to construct an ECM-mimicking hydrogel scaffold by incorporating a polydopamine-modified hyaluronic acid (PDA/HA) complex into a dual-crosslinked collagen (Col) matrix for growth factor-free cartilage regeneration [52]. The authors showed that this scaffold modulated the immune microenvironment and enhanced cartilage regeneration [52]. Zhou et al. [53] created a biomimetic titanium surface using mussel adhesion-mediated ion coordination and molecular clicking strategies. This coating was able to target the integrin α2β1 receptor and inhibit downstream proinflammatory signaling in order to promote osteogenesis and angiogenesis. Mechanical testing for the DPA-Co/GFO group resulted in a greater strength than that of the control group (79.04 ± 3.20 N vs. 31.47 ± 1.87 N, *p* < 0.01), showing that the biomimetic coating matches the natural process for bone regeneration in vivo. Wu et al. [54] engineered a mussel-inspired polydopamine (PDA) coating on a biocompatible porous scaffold, which markedly promoted bone repair and regeneration in a rat calvarial defect model. Lv et al. [55] incorporated PDGF-BB into a chitosan/silk fibroin and created a smart, injectable hydrogel that was thermo-responsive. The hydrogel was designed for an initial burst release of PDGF-BB and continual release of BMP-2, which allowed for highly efficient bone regeneration and a higher bone volume and mineral density in a rabbit model of calvarial defects, demonstrating excellent angiogenic and osteogenic properties. Together, these two models are used in preclinical regeneration studies to further identify directions to test the limits of the immune system.

### 3.3. Hydrogel Testing

This section reviews a wide variety of experiments that have been performed on hydrogel networks. Niu et al. [56] included BMP-2@ZIF-8/PEG-NH2 nanoparticles in a sodium alginate/hydroxyapatite/polyvinyl alcohol hydrogel, loaded it with PDGF-BB, and showed that the hydrogel had a high encapsulation efficiency and long-term bioactivity, good mechanical strength and injectability, which allowed the sequential release of growth factors to promote vascularized bone (calvarial) tissue regeneration. Bai et al. [57] created a 3D inorganic–organic supramolecular bioactive interface that encapsulated bone marrow mesenchymal stem cells (BMSCs) and BMP-2 to demonstrate that the sustained release from the hydrogel induced the osteogenic differentiation of BMSCs and decreased osteoporotic microenvironment around the bioactive interface. Furthermore, it was shown that the 3D inorganic–organic supramolecular bioactive interface can serve as an artificial prosthesis for osteogenesis-deficient patients. Vinikoor et al. [58] created a piezoelectric hydrogel that enhanced cell migration and induced stem cells to secrete TGF-β1, which promoted chondrogenesis as a treatment for osteoarthritis. Specifically, this study exhibited an increased subchondral bone formation and better hyaline cartilage structure. The modulus value after 2 months of these piezoelectric treatments (~5.3 ± 0.3 Gpa) was close to that of healthy native cartilage (6.04 ± 0.4 GPa). Lin et al. [59] developed an injectable PLGA hydrogel that had a sustained release of TGF-β3 and reported that the sustained release of TGF-β3 can improve the biomedical applicability of mPEG-polypepetide scaffolds and promote prolonged articular cartilage regeneration. Nepal et al. [60] loaded BMP-2 onto an oligo-tetra-PEG gel, which demonstrated a sustained release of the growth factor over a 21-day period. Yuan et al. [61] created a combined approach using VEGF and endogenous calcium-capturing gelatin methacrylate hydrogels to promote bone tissue regeneration, and this combined approach was able to simultaneously induce osteogenesis and angiogenesis, necessary processes for fracture repair. Sen et al. [62] demonstrated that adding calcium phosphate to hydrogels allowed for their stiffness to be fine-tuned to a desired range and thus offered a better controlled osteogenic differentiation of embedded human mesenchymal stem cells. Bai et al. [63] used tannic acid as a phenolic glue to combine silk fibroin with hydroxyapatite, and it significantly improved the toughness (by 3.68 morex and adhesion strength (to 922.83 kPa) of the hydrogel by increasing the amount of dissipating energy and allowing for an adequate and stable fixation of the bone fracture. Kim et al. [64] tethered alendronate to an alginate hydrogel to improve bone healing and drug-loading stability and found that the hydrogel had good mechanical stiffness and appropriate stress–relaxation and cell behavior, which indicated that it can be applied for bone tissue engineering. Ali et al. [65] doped 1393 derived glass-based scaffolds that followed the formula of (54.6 − X)SiO_2_·6Na_2_O·7.9 K_2_O·7.7 MgO·22 CaO·1.74 P_2_O_5_ with copper oxide. The copper oxide ensured compatibility, enhanced cell proliferation, and improved the flexural strength and elastic modulus of the hydrogel (Table 1 and Table 2).

### 3.4. Stimulation-Responsive Hydrogels

Stimulation-responsive hydrogels have specific properties including volume, network structure, mechanical strength, or other properties that respond to physical, chemical, and biochemical stimuli. Physical stimuli include temperature, pressure, light, and electric and magnetic fields, while chemical stimuli comprise pH, ionic strength, and chemical agents. In addition, there are biochemical stimuli that involve ligands, enzymes, antigens, and other drugs [66]. Temperature-sensitive hydrogels are commonly used as injectable hydrogels because they are liquid at room temperature and can be gelled within the target tissue at physiological temperatures [67]. While this section discusses physical, chemical, and biochemical stimuli, it is important to recognize thermal stimuli, as the human body maintains a very narrow range of temperatures. Examples include those by Turabee et al. [68], who created a temperature-sensitive Pluronic F127 hydrogel that was embedded with N,N,N-trimethyl chitosan. A sol–gel transition occurred at physiological conditions, which allowed for the sustained release of a chemotherapy drug, docetaxel, to treat glioblastoma. Self-healing hydrogels can autonomously repair structural damage, which allows them to restore tissue’s mechanical integrity and function. The effect of temperature on hydrogels needs to be carefully monitored, as the human body’s internal temperature is significantly higher than room temperature, where the synthesis of these hydrogels typically occurs.

Physical stimuli-responsive hydrogels can alter their behavior in response to external physical prompts, such as light, magnetic fields, or mechanical forces. C. Gao et al. [69] manufactured a temperature- and pH-sensitive Xylan-based hydrogel by crosslinking Xylan with *N*-isopropylacrylamide and acrylic acid and showed that the drug cumulative release rate was 90% and 26% in intestinal and gastric fluids, respectively. The authors suggested that these types of hydrogels could be used as drug carriers for intestinal-targeted oral drug delivery. Tang et al. [70] generated a nanocomposite magnetic hydrogel with dual anisotropic properties, which exhibited direction-dependent behavior depending on applied force. This hydrogel also induced the differentiation of mesenchymal stem cells and osteogenesis, and stimulated bone regeneration by activating the NOTCH1/2 signaling pathways. In the control group the bone volume fraction was 0.2498 ± 0.0671 compared to the magnetic anisotropic hydrogel group, which had a bone fraction volume of 0.5970 ± 0.0374. Sakai et al. [71] created a hydrogel that allowed for the transcutaneous application of carbon dioxide, which increased the pressure of oxygen in tissues due to the Bohr effect, and the authors suggested that it can be used in clinical therapies. These hydrogels have the ability to translate external physical signals into dynamic changes in their activity.

Chemical stimuli allow hydrogel to undergo structural changes that reflect the surrounding chemical environment. Stimuli such as pH, ion concentration, reactive conditions, molecules, and biochemical structures can alter the performance. Sharma et al. [72] synthesized a chitosan and acryloyl-phenylalanine hydrogel that was self-healing, based on hydrogen bonding between the amino group of chitosan and carboxyl group of acryloyl-phenylalanine, and demonstrated a controlled and prolonged release at therapeutic levels of a hydrophobic drug. In addition, it offered good swelling, mechanical properties, biocompatibility, and cell proliferation characteristics. Zhang et al. [73] created a hydrogel constructed from deferoxamine@poly(ε-caprolactone) nanoparticles (DFO@PCL NPs), manganese carbonyl (MnCO) nanosheets, gelatin methacryloyl hydrogel, and a polylactide/hydroxyapatite (HA) matrix and showed that, by upregulating the M2 phenotype of macrophages, it promoted angiogenesis and inhibited osteoclast differentiation, giving it immunomodulatory properties. Wang et al. [74] created a composite hydrogel, embedding CGRP-functionalized polydopamine-coated MXene nanosheets into boronic acid-modified oxidized hyaluronic acid-crosslinked carboxymethyl chitosan. This hydrogel targeted the sensory nerve, which aids in reestablishing the vascular–neural network at the facture site, and it significantly promoted early callus formation and also regenerated the neurovascular network.

Biochemical stimuli create smart hydrogels, designed to react to specific stimuli in vivo. These stimuli are introduced to mimic the natural environment surrounding the bone and to test the limits of the immune system. The immune system is highly selective and has a high sensitivity, making it necessary to test the response of the hydrogel in an ex vivo environment before introducing it to any animal models. Hydrogels can be synthesized or engineered to exhibit responsiveness to a range of physiological stimuli, including pH, ionic strength, and temperature [75]. Wu et al. [76] created an injectable, photocurable biomaterial made from alginate methacrylate, alginate-graft-dopamine, and polydopamine (PDA)-functionalized Ti3C2 MXene nanosheets that allowed for immunomodulation, osteogenesis, and bacterial elimination. The thermal stimulation allows it to attenuate local immune reactions and further promotes new bone formation without the need for exogenous cytokines, cells, or growth factors. Wan et al. [77] created an injectable hydrogel that contained targeting peptide-modified engineered exosomes to be used as therapy for osteoarthritis. The engineered exosomes were loaded with a small inhibitor, LRRK2-IN-1, which inhibited osteoarthritis-related inflammation and immune-related gene expression. Thus, by integrating biochemical molecules with hydrogels, this can bridge the gap between synthetic, laboratory-made scaffolds and structures naturally found in vivo (Table 3).

## 4. Preclinical and Clinical Applications

**Figure 5:**
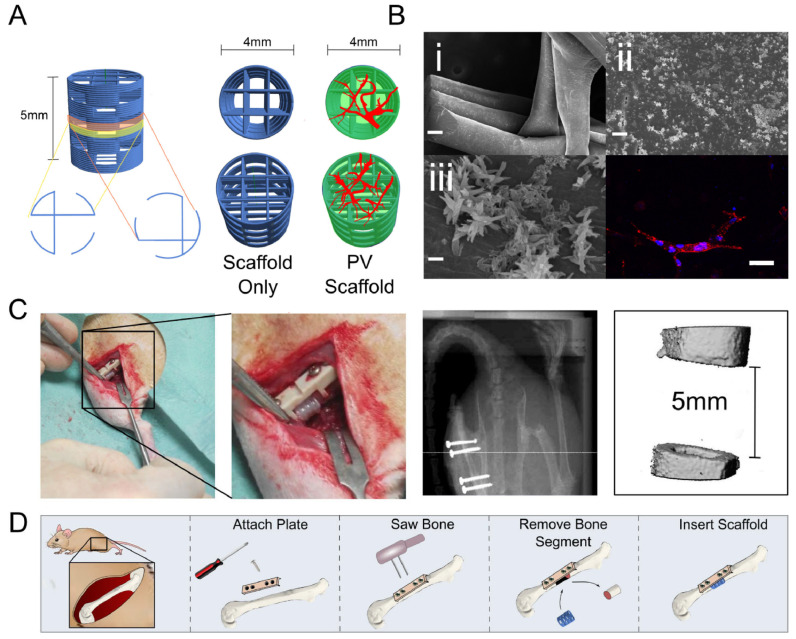
This figure demonstrates how the hydrogel scaffolds were inserted into femoral bone defects of rats. (**A**) Porous PCL scaffold design; (**B**) Images showing nHA coating and micro vessels within scaffolds; (**C**) Images of femoral rat defect location and dimensions; and (**D**) schematic of surgical procedure. Adopted from [79].

There are several preclinical and clinical applications for using hydrogels in bone repair. For example, Nulty et al. [79] demonstrated that implanting hydrogels that were co-cultured with human umbilical vein endothelial cells into critical-size bone defects increased the formation of endothelial cells’ tubular structures and promoted angiogenesis and bone regeneration (Figure 5).

Conley et al. [80] developed a nanohybrid peptide hydrogel that catalyzed scavenging proinflammatory reactive oxygen species and promoted ECM remodeling, while also delivering pro-regenerative cytokines including GDF-5 to suppress immune responses and eventually restoring the regenerative microenvironment of the ECM. Ren et al. [81] demonstrated that a poly(γ-propargyl-l-glutamate) (PPLG) conjugated with azido-modified mannose and 3-(4-hydroxyphenyl) propanamide (HPPA) showed biocompatibility. Chondrocytes encapsulated in this hydrogel showed a spherical morphology, high variability, and high proliferation. Kawaguchi et al. [82] found that implanting a hydrogel containing Fibroblast Growth Factor-2 into a fracture on the midshaft of the right ulna resulted in better mechanical properties, including an almost double increase in maximum load and breaking energy. Minier et al. [83] measured the impact of a calcium phosphate hydrogel loaded with BMP-2 on bone regeneration in a standardized 2 cm long defect in dogs and compared it to using autologous bone grafts. They evaluated the bone healing radiographically at 4, 8, 12, 16, and 20 weeks postoperatively and found that the addition of BMP-2 promoted bone regeneration similar to autologous bone grafts. Gosain et al. [84] implanted hydroxyapatite–ceramic and hydroxyapatite–cement paste into subcutaneous and intramuscular soft tissue pockets in 10 adult sheep, as well as cranial bone graphs of equal dimension for controls. One year after implantation, they confirmed the occurrence of true osteoinduction within the biomaterials and found that the rate of osteoinduction was greatest when a porous architecture was maintained, which occurred better with ceramic than cement paste hydroxyapatite. Fisher et al. [85] treated trochlear chondral defects in mini pigs with microfractures, autologous cartilage transfers, or an acellular hyaluronic acid hydrogel and found that the bony remodeling is likely due to biological phenomena and is not a result of altered mechanical loading. The hydrogel they used was acellular and had no discernable impact on bony remodeling or tissue morphology but could be combined with an exogenous cell source or chemical factors to promote chondrogenesis. The absence of adverse effects suggested that the hyaluronic acid hydrogel was biocompatible in this injury model. Yang et al. [86] reported that, by introducing xonotlite nanofiber Ca_6_(Si_6_O_17_) (OH)_2_ into a 3D-printed fibroin/gelatin basal stimulated bone repair, created a regeneration-friendly osteoimmune microenvironment, and possibly achieved immune reprogramming in macrophages. The studies exhibited that the compressive modulus of the hydrogel was 14.78 ± 0.6108 kPa, which was suitable for the osteogenic differentiation of bone mesenchymal stem cells. Boo et al. [87] used gentamicin-loaded thermoresponsive hydrogels to test fracture healing upon the clearance of *Staphylococcus aureus* in a rabbit model. They reported that the hydrogel was an effective antibiotic carrier and did not impact humeral stiffness or histological scores for fracture healing compared to controls. In the presence of bacterial contamination, rabbits who did not receive the hydrogel had a lower humeral stiffness and higher histopathological scores compared to the group that received the gentamicin hydrogel. This shows the suitability of hydrogels loaded with antibiotics for use in fracture repair (Table 4).

**Figure 6:**
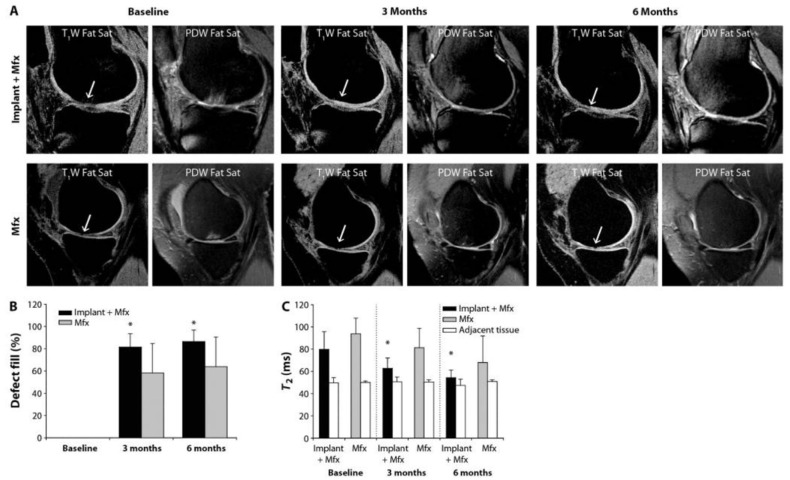
Imaging from a clinical evaluation of cartilage repair. (**A**) Magnetic resonance imaging of cartilage repair with implant and microfracture compared to microfracture alone; (**B**) quantification defect fill; and (**C**) T_2_ relaxation times. * *p* < 0.05. Adopted from [92].

As for clinical studies, Skogh et al. [88] treated defects made during a craniotomy with hydrogels loaded with BMP-2, hydrogels without BMP-2, and Spongostan alone or Tisseel mixed with autologous bone matrix for controls. They assessed bone healing after 3 and 6 months with CT scans and found that defects treated by hyaluronan-based hydrogel showed good healing of cranial defects and were comparable to autologous bone transplants. No local or systemic side effects such as inflammation or excessive bone overgrowth were observed. Niikura et al. [89] used the topical cutaneous application of carbon dioxide via hydrogels to improve fracture repair. Briefly, they enrolled 19 patients with fractures of the femur and tibia, applied a CO_2_ absorption-enhancing hydrogel to the fracture site, and performed transcutaneous CO_2_ absorption therapy. They found no adverse effects, including hypercapnia, and the mean ratio of blood flow 20 min after CO_2_ therapy increased twice as much as the pre-treatment level, leading to the conclusion that CO_2_ therapy using absorption-enhancing hydrogels can enhance blood flow to fractured limbs. Abrahamsson et al. [90] recruited patients with osseous and soft tissue defects on their alveolar process. First, an osmotic soft tissue expander containing a hydrogel was placed under the periosteum, and two weeks later the expander was removed, and a bone graft was placed in the expanded area. The results showed that hydrogel expansion of the periosteum could achieve a surplus of soft tissue to cover bone grafts. Wolf et al. [91] used the ChonDux hydrogel scaffold to treat focal articular cartilage defects in the knee over 24 months. They enrolled 18 patients and found that ChonDux maintained durable tissue restoration, with a final defect percent fill of 94.2% ± 16.3%. The tissues treated with ChonDux had a T2 relaxation time similar to uninjured cartilage, as well as decreased VAS pain scoring and increased IKDC knee function scores. Sharma et al. [92] created a pilot study to measure human cartilage repair using a photoreactive adhesive hydrogel composite. They combined a PEG-based hydrogel with a chondroitin sulfate tissue primer and standard microfracture surgery for 15 patients who had focal cartilage defects on the femoral condyle, while control patients were treated with microfracture alone. The results showed that the treated patients had higher levels of tissue fill (86%) compared to controls (64%) using magnetic resonance imaging, and that the treated patients had less pain (Figure 6).

Matheny et al. [93] conducted a randomized, controlled trial on patients who underwent bilateral total ethmoidectomy to see whether the application of a hyaluronic acid hydrogel compared to carboxymethylcellulose would affect wound healing. It was demonstrated that the hyaluronic acid hydrogel showed a reduction in overall endoscopic grade (*p* < 0.05), synechiae formation, and a trend towards better remucosalization, indicating that the self-crosslinked hyaluronic acid hydrogel provided superior wound healing after ethmoidectomy. Park et al. [94] used a hyaluronate hydrogel that contained human umbilical cord blood-derived mesenchymal stem cells and applied it to the lesion site of osteoarthritic knees for seven patients. They measured the ICRS cartilage repair grade and found improved clinical outcomes throughout 7 years of follow-up using MRI, histology, and a visual analog scale for pain during walking. Kuroda et al. [95] used a gelatin hydrogel containing recombinant human FGF-2 to treat osteonecrosis of the femoral head. A total of 64 patients were enrolled with either nontraumatic, precollapse, or large ONFH, and were administered the gelatin hydrogel. They measured the joint preservation period and found that the radiological and preservation time was higher in the rhFGF-2 group than in the control, and postoperative clinical scores improved significantly. Kawaguchi et al. [96] used a gelatin hydrogel with FGF-2 to treat tibial shaft fractures by enrolling 70 patients. The results indicated that the cumulative percentages of patients with radiographic bone union were higher in the rhFGF-2-treated groups than the placebo group, and that there were no significant differences in adverse events between the groups. Finally, Malizos et al. [97] examined how antibiotic-loaded hydrogel coatings could be used to reduce post-surgical infections after internal osteosynthesis. They enrolled 256 patients who were scheduled to receive osteosynthesis for closed fractures and assigned them an antibiotic-loaded hydrogel coated with Defensive Antibacterial Coating or to a control group. They compared laboratory tests, wound healing, clinical scores, and X-rays and found that six surgical site infections were observed in the control group compared to none in the treated group. In addition, there were no differences in systemic or local side effects nor interference with bone healing related to the DAC hydrogel (Table 5).

### 4.1. Mechanical Properties and Design Parameters for Hydrogels in Load-Bearing Applications

The Hydrogel mechanical strength and design requirements differ significantly between non-load-bearing tissues (dental) and load-bearing bones due to dissimilarities in mechanical and biological demands. In soft tissues such as the pulp, gingiva, and periodontal ligament, hydrogel use focuses on biocompatibility, bioactivity, and controlled degradation over mechanical strength. Natural polymers like collagen, gelatin, hyaluronic acid, and chitosan are favored for their cell adhesiveness and enzymatic degradability, with pore sizes of 50–200 µm, stiffnesses of 0.1–10 kPa, and degradation times of days to weeks, aligning with native soft tissue properties [98]. On the other hand, hydrogels for load-bearing application repair must provide mechanical stability and osteoconductivity under physiological loading. Composite scaffolds combining synthetic polymers (e.g., PEG, PLGA) with inorganic fillers (e.g., hydroxyapatite, bioactive glass) achieve pore sizes of 200–500 µm, moduli from hundreds of kPa to several MPa, and degradation over weeks to months, ensuring the desired structural support during bone regeneration [99] (Table 6).

### 4.2. Safety Profiles for Clinical Translation

When assessing the translational potential of hydrogel scaffolds for drug delivery and tissue engineering, two critical parameters must be considered: (i) safety profiles, including adverse events, biocompatibility, and immune response, and (ii) in vivo degradation and clearance, encompassing the material’s residence time, degradation by-products, and the efficiency of its resorption or elimination. Below, we summarize the current knowledge on these key metrics for hydrogels and highlight several FDA-cleared or FDA-regulated hydrogel scaffolds with well-characterized safety and degradation profiles that serve as benchmarks for clinical translation (Table 7).

## 5. Discussion

Hydrogels are a useful biomaterial for bone repair because of their versality and design flexibility. They also offer tunable mechanical and chemical properties which allow for customization based on application. In addition, the ECM-like structure of hydrogels promotes nutrient/waste transport, cell infiltration, and blood vessel growth. The development of additives and creation of hydrogels responsive to various stimuli have expanded their therapeutic potential for bone tissue engineering by improving the process of osteogenesis and angiogenesis.

While the use of hydrogels for bone repair is very promising, there are some translational barriers, including inflammatory response, lipogenesis, difficulty with dose control, and long-term retention of supplemental growth factors at the injured area [103]. Controlled release is important because bolus delivery can lead to fast clearance of the growth factor and/or drug and off-target effects. Thus, strategies for maintaining controlled release include using smart hydrogels that are responsive to changes in pH, temperature, electric fields, pressure, and the presence of other biomolecules [104]. More research is needed on the long-term immune responses of hydrogels to prevent fibrosis and scaffold rejection. Another area for future research is the combination of hydrogels with gene and stem cell therapy for personalized medicine [105].

Hydrogels must be non-toxic, biocompatible, biodegradable, stable, and be able to support nutrient transport and growth factor delivery [106]. For load-bearing bone fractures, the hydrogel must be able to withstand significant mechanical forces and stresses. This can be achieved by using composite hydrogels with ceramics such as hydroxyapatite, nanomaterials, or fibrous reinforcements. Also, most of the current studies regarding hydrogels use small animals, which do not fully replicate human physiology and loading conditions. More research is needed using large-animal and human clinical trials to demonstrate their efficacy as well as their safety, reproducibility, and long-term outcomes.

One exciting area that warrants future research is the use of artificial intelligence (AI) and machine learning (ML) in driven biomaterials design. AI can assist in predicting and optimizing the properties of hydrogels and material discovery through analyzing and predicting the behavior of materials using the available data to assist researchers in conducting more efficient and targeted experiments [107]. Machine learning (ML) uses algorithms to rapidly analyze large datasets and identify any relationships between biomaterials [108]. These predictions can create opportunities for new biomaterials to be used in novel ways. The combination of AI and ML has the potential to save time and money and may revolutionize how hydrogels are developed and clinically used.

AI algorithms can also simulate the testing of biomaterials used in bone regeneration without having to conduct experiments. Properties such as concentration, crosslinking, kinetics, mechanical strength, and immune system response can be simulated and studied. This insight will allow researchers to digitally sort through various hydrogel parameters without having to conduct lengthy experiments. Deep learning models identify patterns and connections from a previously unknown dataset. Novel biomaterials can be proposed based on a patient’s needs. The algorithm could be trained to tailor the hydrogel via simulation. Amamoto et al. [109] have used a deep learning model to propose new biomaterial designs that are environmentally friendly. AI-driven models could potentially allow laboratories to create patient-specific hydrogels for bone regeneration based on the patient’s health history, microenvironment, and medications. While these technologies are still being developed, undoubtedly, they represent a shift towards more efficient biomaterial creation and testing.

Moreover, there are immunoregulatory challenges that surround the biomaterials being developed and represent a major obstacle. The human immune system is extremely complex and heavily relies on communication. Implanting a hydrogel in vivo will trigger an immunocascade response which may affect the regenerative potential of bone. Researchers are conducting ex vivo tests to ensure that no adverse effects will occur if novel biomaterial is inserted in vivo. These experiments are lengthy and demand extensive analyses before clinical trials proceed. Animal models are another challenge, as there is a wide range of variability. Like the immunoregulatory challenges, animal models are difficult to standardize, thereby slowing down the process of translation to clinical trials. The need for the clinical testing of hydrogels is indeed warranted, since the bottleneck between preclinical translation and clinical translation is a major barrier in regenerative medicine. Regenerative medicine exists to decrease the need for invasive procedures and increase the use of injectable hydrogels that will facilitate not only bone repair but aid in recovery, pain reduction, vascularization, and integration with the native bone tissue. Establishing clear clinical guidelines will be crucial in the transition between laboratory and regenerative studies. Currently, only two trials are listed in clinicaltrials.gov: one study examining the use of the effects of nitroglycerin hydrogel on repairing critical bone defects and the other on the quantitative and clinical assessment of a bioresorbable hydrogel for distal radius fracture repair. The low number of trials highlights the importance between laboratory results and human patients. As such, we hope that many additional studies on the various types of hydrogels are clinically tested, and hopefully some will make it to the market as additional therapeutic treatments for bone repair.

## 6. Conclusions

Hydrogels are a promising alternative to traditional bone grafting strategies because of their customization, biocompatibility, and ability to retain growth factors and additives. While challenges remain with hydrogels regarding their mechanical strength as well as their immune interactions and large-scale clinical use, an interdisciplinary approach that combines cell and molecular biology, biomedical engineering, material science, and orthopedics will help overcome these challenges. Hydrogels have the potential to change how we approach complex bone injuries and can significantly improve patient outcomes and reduce the emotional and economic burden.

## Figures and Tables

**Table 1:** Growth factors in bone repair.

Hydrogel	Growth Factor	Outcome	Growth Factor Concentration	Release Kinetics	Reference
MgFe-LDH Nanosheet	PDGF-BB	Thermo-responsive, burst release of PDGF-BB, sustained release of BMP-2		Burst release of PDGF-BB	[55]
GelMA (gelatin methacryloyl)	BMP-2	Stronger osteogenic induction capacity	Exosome loading of 500 μg/mL in hydrogel	GM-90-CP05 group released exosomes for the longest duration	[44]
Supramolecular polysaccharide		Osteogenic differentiation of bone marrow mesenchymal stem cells	50 μg BMP-2 was encapsulated into 600 μL N-chitosan and ADH	No initial burst release during the first day, and about half of growth factor released within a week (48.7 ± 2.1%)	[57]
Oligo-tetra-PEG-gel		Bone regeneration of critical-sized calvarial defects within 28 days	For every 20 g mouse body weight, 2 μg of rhBMP-2 dose	Sustained release of rhBMP-2 over 21 days	[60]
Sodium alginate/hydroxyapatite/polyvinyl alcohol hydrogel	PDGF-BB and BMP-2	Vascularized bone regeneration	Not reported in text	Burst release of PDGF-BB and sustained release of BMP-2	[56]
PLG-*g*-TA/VEGF/Sr-BGNPs	VEGF	Increased proliferation and osteogenic differentiation of rat bone marrow mesenchymal stem cells	5% (*w*/*v*) of the PLG-g-TA copolymer, 8 units/mL of HRP, and H_2_O_2_; VEGF loading not reported	Biphasic initial explosive release and subsequent slow release	[45]
Endogenous calcium-capturing gelatin methacrylate		Induced simultaneous osteogenesis and angiogenesis	1 mg BP + 10 mg PLA/G@CP short fibers per 10 mL GelMA solution (BP stabilizes VEGF)	Initial burst then sustained release for 18 days	[61]
Collagen matrix	TGF-β1	Increased chondrogenesis	NF-sPLLA, 1, 5, and 10 mg/mL, rat tail collagen I solution (3 mg/mL in acetic acid); concentration of TGF-β1 not reported	Gene expression present after 2 days; in vivo effect after 2 months	[58]
Poly(lactic-co-glycolic acid) (PLGA)	TGF-β3	Modification of biosynthetic and differentiation activities of chondrocytes		Sustained release of TGF-β3 through microspheres	[59]

ADH (antidiuretic hormone), BMP (Bone Morphogenetic Protein), GelMA (gelatin methacryloyl), HRP (horseradish peroxidase), PDGF-BB (Platelet-derived Growth Factor-BB), TGF-β (transforming growth factor-beta), VEGF (Vascular Endothelial Growth Factor).

**Table 2:** Examples of functionalized hydrogels and their use.

Hydrogel	Additive	Function	Outcome	In Vitro Findings	In Vivo Findings	Reference
Gelatin and hyaluronic acid methacrylate	Stem cell exosomes	Osteogenesis	Accelerated bone regeneration	CCK8 assay demonstrates biocompatibility	BMSC promoted osteogenesis, and EPCs promoted angiogenesis	[43]
Gelatin sponge	Superparamagnetic iron oxide nanoparticles	MRI contrast agent for T2-weighted MRI	Induce active osteogenesis	N/A	Micro-CT and histology results show improved bone formation	[46]
3D-printed polycaprolactone scaffold, aldehyde hyaluronic acid, and carboxymethyl chitosan	Mesoporous silica nanoparticles containing fingolimod, vancomycin	Dual-drug delivery	Infection control and bone regeneration	FTY720-loaded scaffold has excellent biocompatibility	Rat model: composite scaffold	[47]
Nanocomposite	Piezoelectric nanoparticles	Controllable electrical output	Enhanced osteogenic effect	Adhesion experiment showed good stability and satisfactory adhesion; great biocompatibility	High-efficiency bone adhesion properties (3-fold strength increase)	[48]
Chitosan gelatin	Cerium oxide nanoparticles	Scavenge free radicals	Highly biocompatible, can be applied to bone repair	79% porosity, 45.99% weight loss, Cyto-friendly to MC3T3-E1 cells, biocompatible	N/A	[49]
Chitosan/PVA	Hydroxyapatite	Surface mineralization	Excellent tensile strength of 0.24 MPa, rapid bone defect repair	N/A	Accelerated bone regeneration in rabbit bone defect model	[50]
Polyethylene glycol	Muscle-derived biomimetic coating	Regulation of macrophage differentiation	Immunomodulation and vascularized bone regeneration	Biocompatibility demonstrated via LIVE/DEAD cell staining procedure	Vascularized bone regeneration at bone-implant surface	[53]
Agarose and agarose–collagen blend	Calcium	Major role in bone mineralization	Increased range of hydrogel stiffness	Increased H-type vessel (found in bone marrow); hydrogel supports cells, biocompatible	N/A	[62]
Alendronate-tethered alginate		Osteogenesis	Promoted proliferation and differentiation	Demonstrated osteogenic differentiation 3–6 times more than control; assays report increased cell viability	N/A	[64]
Silf fibroin and hydroxyapatite	Tannic acid	Phenolic glue	Improved toughness and adhesion strength	Biocompatible	Stable fixation of bone fracture	[63]
1393 scaffold	Copper oxide	Stimulant for bone formation	Greater cytocompatibility, cell proliferation	1393 scaffold with concentration 5 mg/mL had viability of 97.80%; enhanced cellular proliferation	N/A	[65]

MC3T3 (mouse preosteoblast cell line), Micro-CT (micro-computed tomography), MRI (magnetic resonance imaging), PVA (polyvinyl alcohol).

**Table 3:** Hydrogel special properties.

Hydrogel	Special Property	Outcome	In Vitro Findings	Reference
Xylan-based *N*-isopropylacrylamide-co-AA	Temperature and pH sensitive	Intestinal-targeted oral drug delivery	Biocompatible with NIH3T3 cells	[69]
Polyethylene glycol diacrylate-methacrylated gelatin	Magnetic	Bone tissue regeneration	Biocompatibility with BMSCs	[70]
Sodium alginate–methylparaben–sodium dihydrogen phosphate	Validation of the Bohr effect	Decreased intracellular pH of the triceps surae muscle	Enhances CO_2_ absorption	[71]
Chitosan and acryloyl-phenylalanine	Self-healing	Good mechanical properties	No cytotoxicity for HEK-293 cells up to 200 μg/mL	[72]
Gelatin methacryloyl	Osteoimmunity-regulating	Superior osteogenic abilities	Reported biocompatibility	[73]
Boronic acid-modified oxidized hyaluronic acid-crosslinked carboxymethyl chitosan	Sensory-nerve targeting	Improved callus mineralization	CCK-8 and LIVE/DEAD assays show cell viability on days 1, 4, and 7	[74]
Alginate methacrylate, alginate-graft-dopamine, and PDA functionalized Ti3C2 Mxene nanosheet	Controllable thermal stimulator	Promotes new bone formation	With mild heating conditions supports cell growth	[76]
Gelatin methacryloyl hydrogel	Pepetide-modified engineered exosomes	Osteoarthritis therapy	No inhibition of chondrocytes (0.5, 1.0, 2.5, and 5.0 μM)	[77]
Hydroxyapatite and gelatin methacryloyl	Antibacterial	Preventing bacterial infection	Overall low toxicity and good biocompatibility to MC3T3 cells	[78]

BMSCs (bone marrow mesenchymal stem cells), MC3T3 (mouse preosteoblast cell line), PDA (polydopamine), Ti3C3 (titanium carbide).

**Table 4:** Examples of preclinical applications of hydrogels.

Hydrogel Type	Study Model	Outcome	Inflammatory Markers	Healing Time	Cell Viability	Reference
Fibrin-based with 3D-printed polycaprolactone scaffold	Rat model	Enhanced vascularization, higher levels of new bone formation	N/A	Experimental time: 12 weeks	>70%	[79]
Nanohybrid peptide hydrogel	Rat nucleotomy model	Structural and functional recovery of intervertebral disk				[80]
PPLG-g-Man/HPPA Glycopolypeptide	Subcutaneous model of nude mice	Encapsulated chondrocytes showed spherical phenotype with high viability and proliferation	Minimal in vivo inflammation (histological analysis)	Hydrogels degraded week 4	High viability of chondrocytes	[81]
Gelatin	Nonhuman primate	Accelerated fracture healing	Local inflammation	Median bone union time was 128 days	N/A	[82]
Calcium phosphate	Critical-size segmental defect model of non-union in dogs	Induced formation of well-differentiated mineralized lamellar bone	N/A	20 weeks	Histological analysis shows viable osteocytes	[83]
Hydroxyapatite and beta-tricalcium phosphate	Sheep	Osteoinduction within hydroxyapatite-derived biomaterials	N/A	Experimental time: 12 months	No difference in bone morphology	[84]
Hyaluronic acid	Trochlear chondral defect in Yucatan minipigs	Biocompatibility	Mild inflammation	Experimental time: 6 weeks	Robust staining of chondrocytes	[85]
Xonotlite nanofiber in 3D-printed silk fibroin/gelatin basal scaffold	Rabbit model	Accelerated vascular bone regeneration	VEGF and TGF-β increased, IL-1β decreased	Experimental time: 12 weeks	LIVE/DEAD assay showing cell viability day 21	[86]
poly(N-isopropylacrylamide) grafted hyaluronic acid	Rabit humeral osteotomy	Accelerated fracture healing				[87]

HPPA (3-(4-hydroxyphenyl)propionic acid), IL (interleukin), pAAM (polyacrylamide), PPLG (poly(γ -propargyl-L-glutamate)), TGF-β (transforming growth factor beta), VEGF (vascular growth factor).

**Table 5:** Clinical applications.

Hydrogel Type	Bone Model	Outcome	Quantitative Outcomes Results	Healing Time	In Vitro Findings	Reference
Hyaluronan	Critical-size cranial defects	Bone healing significantly increased				[88]
CO_2_ absorption-enhancing hydrogel [71]	Femur and tibia fractures	Improved fracture repair	20-min exposure increased blood flow at fracture site	N/A	Safe trial in all patients, no toxicity	[89]
Vinyl pyrrolidone and methylmethacrylate	Osseous and soft tissue defect on alveolar process	Soft tissue expansion of the periosteum	Implant tissue expansion 2.3 ± 2.1 mm		N/A	[90]
ChonDux	Femoral condyle defect	Stable restoration of full thickness articular cartilage defects	IKDC scores improve by 30 points	24 months	No adverse effects	[91]
Poly(ethylene glycol) diacrylate		Decreased overall pain severity and frequency	IKDC scores for implant decreased over 6 months	12 weeks	No adverse effects	[92]
Hyaluronic acid	Ethmoid cavity	Reduction in endoscopic grade and synechiae formation; increased wound healing	Statistically significant reduction in endoscopic score	12 weeks	No side effects	[93]
Hyaluronate hydrogel	Osteoarthritic knee	Articular cartilage repair	VAS score decreased and IKDC score increased	24 weeks	Mild swelling	[94]
Gelatin	Osteonecrotic femoral head	Increased joint preservation time	Median joint preservation time in control was 45.8 months, and in experiment was 11.1 months	24 months	In 18.8% of patients, there were adverse effects	[95]
Gelatin	Tibial shaft fracture	Higher percent radiographic bone union	Bone union percentages were higher in treated group	24 weeks	Safe	[96]
Hyaluronic and polylactic acid (DAC)	Closed fracture	Reduced post-surgical site infections	4.6% of control patients had infections, 0% in treated group	6–12 weeks	Safe for use	[97]

DAC (Defensive Antibacterial Coating), IDKC (International Knee Documentation Committee), VAS (visual analog scale).

**Table 6:** Mechanical properties and design considerations for hydrogels.

Parameter	Non-Load-Bearing Sites	Load-Bearing Regions	Design Considerations
Hydrogel Types	Natural polymers (collagen, gelatin, chitosan, hyaluronic acid)	Synthetic/inorganic hybrids (PEG-HA, PEG-bioactive glass, PLGA-hydroxyapatite)	Natural for bioactivity; composites for strength and osteoconductivity
Representative Tissues	Dental pulp, gingiva, periodontal ligament, mucosa	Cortical and trabecular bone, long bone defects	Distinct biological and mechanical environments determine scaffold requirements
Pore Size	50–200 µm	200–500 µm	Smaller pores promote cell attachment and soft tissue ingrowth; larger pores enable vascularization and mineralization
Mechanical Properties	0.1–10 kPa	0.1–5 MPa	Matches native tissue stiffness; bone scaffolds require higher load-bearing capacity
Degradation Rate	Days to a few weeks	Weeks to several months	Degradation tuned to tissue regeneration rate, rapid for soft tissue, slower for bone
Biological Functionality	High bioactivity and cell affinity; minimal mechanical load	High mechanical integrity and osteoconductivity under load	Reflects biological and mechanical priorities of each site
Desired Outcomes	Soft tissue repair, angiogenesis, pulp and periodontal regeneration	Bone formation, mineralization, long-term mechanical support	Functional integration and matched degradation-to-regeneration dynamics

**Table 7:** FDA-cleared hydrogel scaffolds.

Device	Scaffold Role	Degradation Rate (In Vivo)	Safety Profile	Reference
SpaceOAR™ Hydrogel	Perirectal spacer (prostate RT)	~12 weeks space; absorbed by ~6 months	Post-market: 981 AEs (2015–2023)/~206 k devices; ~13 % grade ≥ 3; majority mild	[100]
DermiSphere® hDRT	Dermal regeneration scaffold (full-thickness wounds)	No data available	FDA 510(k) cleared; safety acceptable; long-term data limited	[101]
Premvia™ Hydrogel Tissue Substitute	Wound care scaffold (partial-/full-thickness wounds, ulcers)	No data available	FDA cleared; publicly available safety/degradation data limited	[102]

## Data Availability

Not Applicable.

## Abbreviations

ADH	Antidiuretic Hormone
BMP	Bone Morphogenetic Proteins
BMP-2@ZIF-8/PEG-NH2	Bone Morphogenetic Protein-2@ Zeolitic Imidazolate Framework-8/Polyethylene Glycol Terminated with Amine Groups
BMSC	Bone Marrow Mesenchymal Stem Cells
MC3T3	Mouse Preosteoblast Cell Line
CGRP	Calcitonin Gene-Related Peptide
DAC	Defensive Antibacterial Coating
DFO@PCL NPs	Deferoxamine@poly(Ɛ -caprolactone) Nanoparticles
DPA-Co/GFO	DPA (3,4-dihydroxy-L-phenylalanine)-Cobalt/The Peptide Sequence GFO (GFOGER)
ECM	Extracellular Matrix
FGF	Fibroblast Growth Factor
GDF	Growth Differentiation Factor
GelMA	Gelatin Methacryloyl
HA2	Hyaluronic Acid Oligosaccharide
HA2-ZOL	Hyaluronic Acid Oligosaccharide–Zoledronate
HPPA	3-(4-hydroxyphenyl)propionic Acid
HRP	Horseradish Peroxidase
ICRS	International Cartilage Repair Society
IL	Interleukin
IGF	Insulin-like Growth Factor
IKDC	International Knee Documentation Committee
IPN	Interpenetrating Polymeric
LRRK	Leucine-Rich Repeat Kinase
MnCO	Magnese Carbonyl
MPa	Mega-Pascals
MRI	Magnetic Resonance Imaging
PCL	Polycaprolactone
PDGF	Platelet-like Growth Factor
PAAM	Polyacrylamide
PDGF-BB	Platelet-like Growth Factor, Two Subunits B
PEG	Polyethylene Glycol
PGA	Polyglycolic Acid
PDA	Polydopamine
PLGA	Poly(lactic-co-glycolic acid)
PLLA	Polylactic Acid
PPLG	Poly(γ -propargyl-L-glutamate)
PVA	PolyVinyl Alcohol
rhFGF	Recombinant Human Fibroblast Growth Factor
SLS	Selective Laser Sintering
SPIONS-GS	Superparamagnetic Iron Oxide Nanoparticles (SPIONs)–Gelatin Sponge (GS)
TGF-β	Transforming Growth Factor Beta
Ti3C3	Titanium Carbide
VAS	Visual Analog Scale
VEGF	Vascular Growth Factor
μCT/Micro-CT	Micro-Computed Tomography
